# Photodynamic Therapy in Cancer: Principles, State of the Art, and Future Directions

**DOI:** 10.3390/pharmaceutics16121564

**Published:** 2024-12-06

**Authors:** Dmitri V. Krysko, Irina V. Balalaeva, Tatiana A. Mishchenko

**Affiliations:** 1 Cell Death Investigation and Therapy Laboratory, Anatomy and Embryology Unit, Department of Human Structure and Repair, Faculty of Medicine and Health Sciences, Ghent University, 9000 Ghent, Belgium; 2 Cancer Research Institute Ghent, 9000 Ghent, Belgium; 3 Institute of Biology and Biomedicine, Lobachevsky State University of Nizhny Novgorod, 603022 Nizhny Novgorod, Russia

## 1. Introduction

Since its discovery more than 100 years ago, photodynamic therapy (PDT) has become a potent strategy for the treatment of many types of cancer [1,2]. This cancer treatment strategy is based on photoactive dyes (photosensitizers, PSs) that, when exposed to visible or near-infrared (NIR) light, trigger the production of cytotoxic molecules, such as reactive oxygen species (ROS). The primary advantage of PDT over conventional chemotherapy lies in its dual selectivity for lesions, achieved through the preferential accumulation of PSs in tumor tissue and the localized application of activating light. Initially, conventional PDT was restricted to superficial tumors or tumors accessible with an endoscope. However, advancements in light delivery systems have broadened the applicability of PDT, enabling its use in treating large solid tumors as well as tumors located deep within parenchymal organs or the brain.

To date, several groups of PSs have been clinically approved for anti-cancer PDT, and an even longer list of photoactive dyes with diverse properties is currently undergoing preclinical and clinical trials [1,2]. The ultimate aim of medicinal chemists is to develop an “ideal” drug that fulfills all the requirements set by clinicians. The most critical requirements include high photodynamic activity, the absence of toxicity in the absence of light, and the highest possible selectivity for tumor tissue. Continuous efforts to achieve this goal result in the synthesis and investigation of hundreds of new molecules each year. We would like to highlight a relatively new concept in this field: the administration of PSs as prodrugs with minimal initial biological activity [3]. The activation of pre-PSs can be light-dependent; in such cases, the triple selectivity comprising selective pre-drug delivery, its localized activation, and the execution of PS function under focused irradiation provides enhanced protection to normal tissues and organs while minimizing side-effects. Another approach involves utilizing tumor-targeting vectors or carriers to maximize the contrast in PS accumulation in tumor and normal tissues. Such photoactive molecules can be conjugated with peptides, antibodies, and low-molecular-weight targeting moieties, or they can be incorporated into nanoscale carriers designed for targeted delivery to tumors [4]. The nanoparticle-based delivery agents allow for the combination of multiple activities, thereby reinforcing the effects of PSs through the co-delivery of drugs with different mechanisms of action or by mitigating tissue hypoxia [2,5].

In addition to advancements in medicinal chemistry, progress in cell biology has played a crucial role in the development of PDT. Recent research has elucidated many aspects of cellular responses to PDT, shifting the perception of PDT from merely an oxidative stress inducer to a more nuanced understanding of the complex molecular mechanisms governing cell death and survival during PDT treatment. The accumulated data have provided at least partial understanding that cancer cells treated with PDT decide whether to survive or undergo cell death based on multiple factors, including their genetic and metabolic features, primary intracellular targets of ROS action, the intensity of PDT exposure, and the surrounding environmental context [6,7,8,9]. The initial concept of PDT-induced cell death occurring solely through necrosis or apoptosis has been significantly modified and expanded to include a broad spectrum of potential outcomes, such as necroptosis, ferroptosis, pyroptosis, and other cell death modalities, each with immunogenic or non-immunogenic properties [10]. This revision has significant consequences for practical anti-cancer therapy, as it offers a way to overcome the frequently observed resistance of tumor cells to apoptosis by redirecting them toward alternative cell death. One of the best examples is the synergistic effect of ferroptotic inducers and PDT in the eradication of tumor cells [11,12].

Moreover, over the past decade, it has become clear that the efficacy of PDT largely relies on its ability to induce immunogenic cell death (ICD), which activates the immune system to generate effective anti-cancer immunity with long-lasting immunological memory [2,7,9,13,14,15]. The ability of various PSs to induce ICD differs and depends not only on the physicochemical properties of the drug but also on its mode of cellular entry—whether individually or within a carrier—as well as on the PDT intensity, which is determined by the combination of PS concentration and light power [2,10]. Summarizing the latest findings in cancer biology, the concept of an “ideal” PS must now include new requirements, such as the ability to overcome cell resistance by inducing different cell death pathways, with a particular emphasis on triggering ICD [10].

Expanding our understanding of the mechanisms underlying cellular responses to PDT has provided a rational foundation for combining PDT with other cancer treatment modalities, particularly immunotherapy and targeted therapy. Additionally, the efficacy of PDT can be enhanced through the combinational administration of chemical compounds that modulate tumor cell sensitivity to PDT, an approach already proven effective in radiation therapy and chemotherapy and now being tested for PDT.

## 2. Overview of the Published Articles

This Special Issue offers insights into recent advancements in understanding the mechanisms of PDT and, building on this knowledge, the development of novel PDT agents, protocols, and strategies. It includes four original articles and one review, each exploring various aspects of designing next-generation PSs and optimizing highly effective PDT protocols.

The use of intelligent photosensitive systems represents an exciting and rapidly evolving trend in PDT. The state of the art in this field is comprehensively discussed in the review by N.S. Kuzmina et al. (Contribution 1). The authors analyze the recent progress in the synthesis and application of intelligent agents that are activated through light-induced redox reactions or the disruption of photosensitive bonds. Particular attention is given to the targeted delivery and activation of PSs using bio-orthogonal chemistry techniques.

The joint efforts of research teams from Germany and the Netherlands, led by S. Oliveira and H. Kolmar (Contribution 2), have resulted in the development of a novel targeting system for fluorophores or PSs based on nanobody dextran polymer conjugates known as dextraknobs. Using PDT based on dextraknobs labeled with the IRDye700DX^®^ PS, the authors demonstrated that these nanobody conjugates enable deep and homogenous penetration into human epidermoid carcinoma A431 tumor spheroids, resulting in the development of a cytotoxic specificity. These results highlight the significant potential of this innovative system for application in cancer diagnostics and therapy.

The study by L. Hübinger et al. (Contribution 3) critically examines the potential of Cherenkov light, generated within tissues exposed to ionizing radiation (IR), to activate PSs. Their accurately performed study revealed a discrepancy between their experimental results and a previously published report [16]. The study found no significant contribution of Cherenkov light to photodynamic effects of two different PSs from the furanocoumarin group in several cell lines subjected to either gamma photon beam or rhenium-188 radionuclide. The authors emphasize the need for further research to identify the factors influencing these observed phenomena.

Extensive in vitro and in vivo studies by T. Redkin et al. focus on enhancing the therapeutic potential of PDT as a foundation for developing breakthrough approaches in immunotherapy of aggressive brain tumors, such as glioblastoma multiforme (Contribution 4). The authors reveal that two novel PSs from the tetracyanotetra(aryl)porphyrazine group (pz I and pz III) act as potent inducers of ICD upon the light irradiation of murine glioblastoma GL261 cells loaded with these PSs. The GL261 cells pre-treated with pz-I-PDT or pz-III-PDT demonstrate strong protection against tumor growth in a heterotopic subcutaneous glioblastoma mouse model. Additionally, their lysates effectively prime dendritic cell vaccines, leading to the activation of adaptive anti-tumor immunity in an intracranial orthotopic tumor mouse model.

The vulnerability of photoinduced activity in tumors to changes in the lipid microenvironment has become an area of emerging interest for enhancing the therapeutic benefits of PDT. The study by M. Korbelik (Contribution 5) presents a proof-of-principal study demonstrating the potential use of anti-tumor lipids as adjuvants to improve PDT efficacy. Using a subcutaneous mouse model of three solid tumors (SCCVII squamous cell carcinoma, MCA205 fibrosarcoma, and Lewis lung carcinoma), the study shows that an elegant combination of peritumoral administration of edelfosine, a synthetic ether lipid with strong ICD properties, and Photofrin-based PDT resulted in at least a doubling of the tumor cure rates in mice.

## 3. Conclusions and Future Directions

PDT has made significant progress over the years, with remarkable new trends emerging in recent decades. The efforts of medicinal chemists and photobiologists have led to significant progress in addressing the limitations of PDT, including insufficient selectivity in the delivery of photoactive dyes (i.e., PS) to tumor tissue, unwanted side-effects, tumor cell resistance, and the limited depth of light penetration into tissue. The new PDT regimens under development leverage the growing understanding of the molecular and cellular mechanisms underlying tumor responses to PDT to maximize the effectiveness of this cancer treatment modality. It is expected that the scope of PDT applications, both as a standalone treatment and in combination with other therapies, will continue to expand.
